# The lung microbiome and asthma: from inflammatory phenotype differentiation to microbe-based immunomodulatory strategies

**DOI:** 10.3389/fimmu.2026.1901065

**Published:** 2026-08-14

**Authors:** Sanguo Ren, Hanle Li, Zeyu Dong, Min Fang

**Affiliations:** 1 School of Life Sciences, Henan University, Kaifeng, Henan, China; 2 Henan Key Laboratory of Synthetic Biology and Biomanufacturing, Henan University, Kaifeng, Henan, China

**Keywords:** asthma, eosinophil, inflammatory phenotypes, lung microbiome, neutrophil

## Abstract

As a complex airway inflammatory disease, the pathogenesis of asthma is closely associated with dynamic changes in the lung microbiome and immune regulation. Asthmatic patients with distinct inflammatory phenotypes exhibit significant alterations in the diversity and dominant microbiota of their lung microbiome, particularly characterized by the colonization of bacteria such as *Haemophilus influenzae*, *Moraxella catarrhalis*, and *Tropheryma whipplei*. The microbial community modulates the differentiation of CD4^+^ T-cell subsets and activates or inhibits inflammatory pathways, thereby exerting interactive effects on the eosinophilic and neutrophilic inflammatory phenotypes of asthma. Microbial products, such as lipopolysaccharide (LPS), exosomes, and bacterial lysates, play crucial roles in activating or suppressing host immune cells, mediating the initiation and progression of airway inflammation. Current studies have primarily elucidated the mechanisms by which the lung microbiome modulates asthma immune regulation and explored immunomodulatory strategies targeting microorganisms and their products, including the potential of bacterial lysates, probiotic therapy, and microbial exosome therapy in asthma. Unlike previous reviews focusing on the gut–lung axis, this review establishes correspondences between specific bacterial species and distinct asthma endotypes, and critically evaluates the methodological heterogeneity in this field. These reflections provide a theoretical basis for standardizing microbiome research, elucidating microbiota-driven differential progression of asthma subtypes, and advancing microbiome-based precision prevention strategies.

## Graphical Abstract

**Figure:**
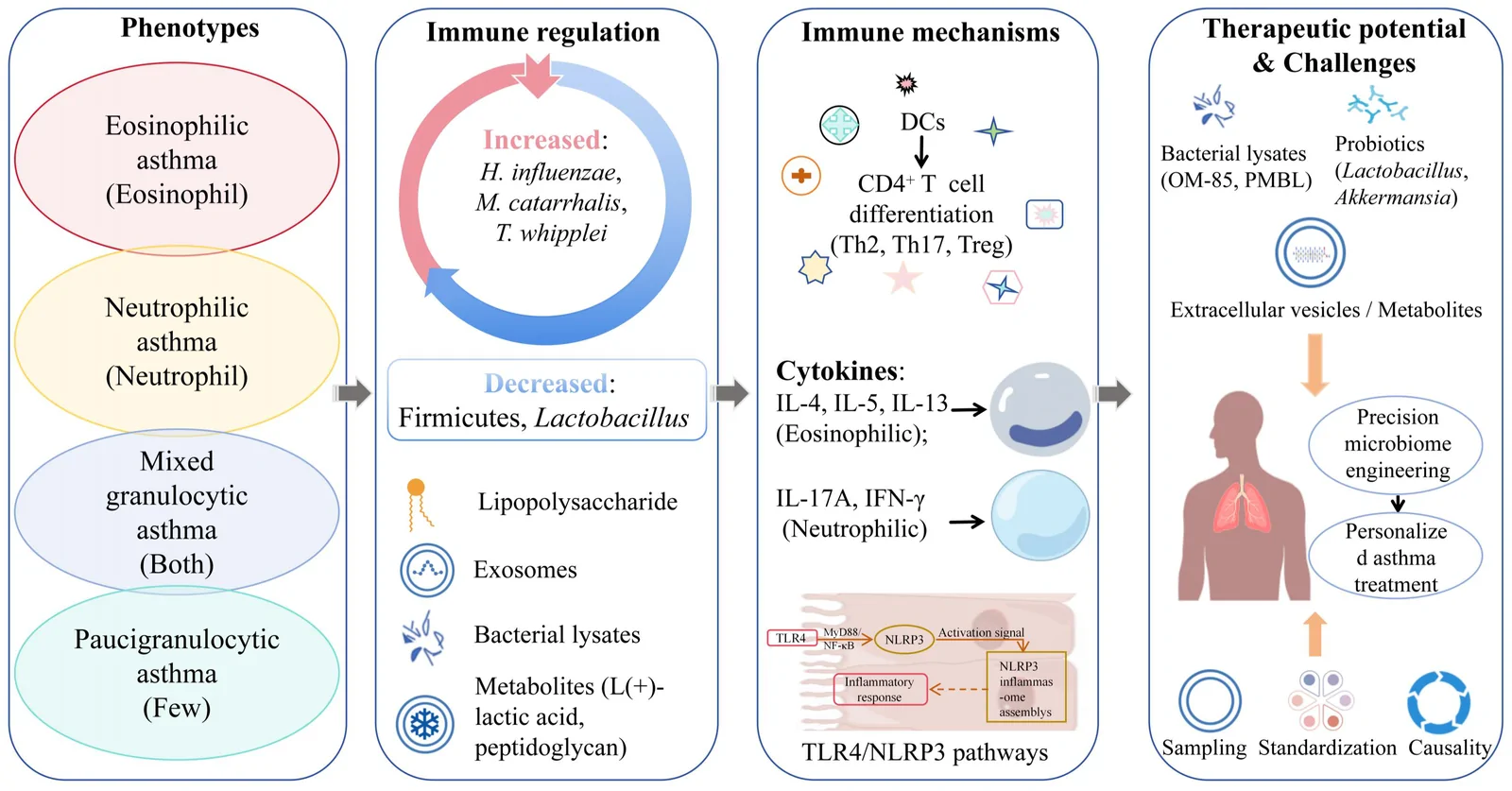
Mechanistic model of lung microbiome–host crosstalk in asthma. Asthma inflammatory phenotypes, eosinophilic, neutrophilic, mixed, and paucigranulocytic, associate with distinct microbiome shifts (enriched *Haemophilus*/*Moraxella*, depleted *Lactobacillus*). Microbial products (LPS, extracellular vesicles) engage host PRRs on dendritic cells, driving CD4^+^ T-cell polarization (Th2/Th17/Treg) and downstream cytokines (IL-4/5/13, IL-17A) and TLR4/NLRP3 pathways, which determine phenotype outcome. Bacterial lysates, probiotics, exopolysaccharides, and metabolites enable phenotype-targeted immunomodulation, advancing precision microbiome engineering; however, standardization and causality challenges persist.

## 1 Background

**Figure 1:**
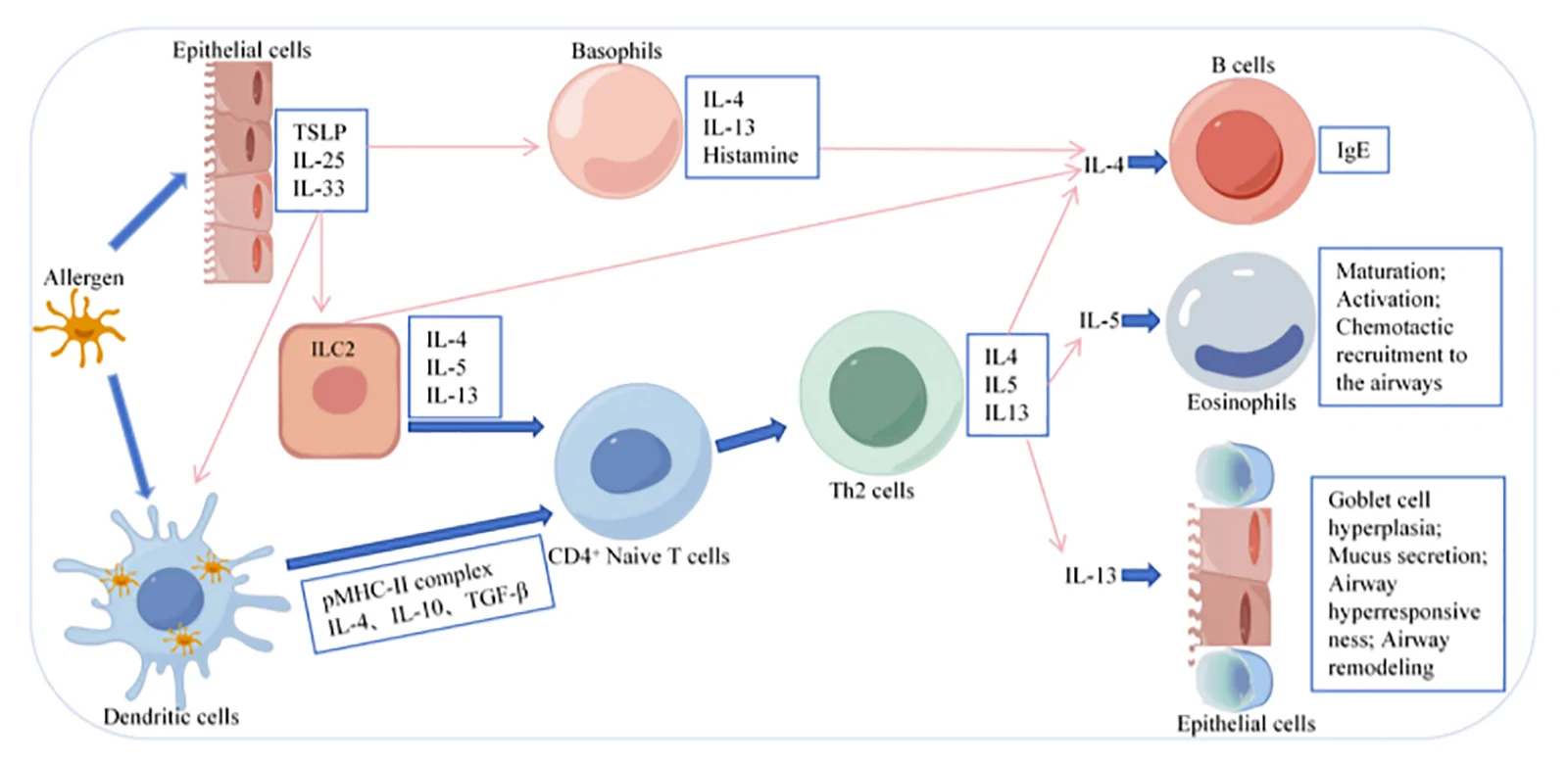
The traditional model of asthma. DCs present allergens to naive CD4^+^ T cells, driving differentiation into Th2 cells and secretion of IL-4, IL-5, and IL-13, which promote eosinophilic inflammation, goblet cell hyperplasia, and AHR. Thin brownish-yellow lines denote promotion, whereas thick blue lines signify functional effects.

Endotype and phenotype represent the two core dimensions of asthma heterogeneity: endotypes are defined by pathophysiological mechanisms (e.g., immune pathways, inflammatory mediators, and biomarkers), whereas phenotypes focus on clinical traits, including inflammatory cell types and treatment responses. A clear distinction between these concepts is essential for unraveling asthma pathogenesis and enabling personalized therapeutic approaches. Asthma, a global chronic respiratory disease, is characterized by airflow limitation caused by airway narrowing, airway wall thickening, and increased mucus production (1). Traditional studies on the pathogenesis of asthma consider it essentially a single allergic inflammatory disease dominated by T helper 2 (Th2) responses (**Figure 1**). Upon allergen stimulation, dendritic cells (DCs) present antigens to naive T cells (T_N_), promoting Th2 differentiation. Activated Th2 cells secrete Interleukin-4 (IL-4), Interleukin-5 (IL-5), and Interleukin-13 (IL-13): IL-4 induces B cells to produce allergen-specific IgE and sensitizes mast cells; IL-5 recruits and activates eosinophils; and IL-13 drives airway hyperresponsiveness (AHR), mucus secretion, and airway remodeling (2). Asthma is a heterogeneous syndrome with overlapping clinical features but distinct mechanisms. Stratification by molecular pathways, inflammatory profiles, and biomarkers defines T2-high versus T2-low phenotypes based on airway expression of IL-13-induced genes and Th2 pathway activation (3). Th2-type endotypes involve Th2 cells, Group 2 innate lymphoid cells (ILC2s), IgE-producing B cells, and eosinophils, whereas non-Th2 endotypes include T helper 17 (Th17), neutrophilic, and paucigranulocytic subtypes. Mixed granulocytic and paucigranulocytic asthma involve concurrent or alternating activation of both Th2 and non-Th2 pathways (4). Asthma management has shifted from treating the disease to addressing identifiable and treatable traits, such as elevated IgE and eosinophilia (5, 6).

Traditional views held healthy lungs as sterile, but culture-independent methods (16S rRNA sequencing, metagenomics) have revealed a low-biomass yet diverse microbial community, overturning this notion (7). This discovery has reshaped the understanding of pulmonary physiology and opened new avenues for investigating the pathophysiology of asthma. Lung microbiome homeostasis is critical for local immune balance and airway barrier integrity; dysbiosis promotes asthma through multiple mechanisms (8), including direct/indirect regulation of host immune cells by microbes or their metabolites, signaling via microbial-associated molecular patterns (MAMPs) and pattern recognition receptors (PRRs), and complex metabolic crosstalk (9, 10). Elucidating these intricate associations holds significant scientific and clinical value for understanding asthma pathogenesis, identifying novel biomarkers, and developing innovative therapies.

This review first elaborates the immunopathological foundations of innate and adaptive immunity in asthma, and further dissects how pulmonary dysbiosis mediates the initiation and progression of distinct asthma clinical subtypes via specific molecular crosstalk. Unlike previous reviews focusing on the gut–lung axis at a macro level, this review specifically addresses species-level differences, profiles the roles of distinct bacterial species across asthma endotypes, and critically examines methodological heterogeneity and current limitations in asthma microbiome research. This work aims to bridge existing gaps in the literature and provide a theoretical foundation for mechanistic exploration and clinical translation of the asthma microbiome.

## 2 Immune microenvironment and pathophysiological basis of asthmatic airways

### 2.1 Functional differentiation of key innate and adaptive immune cells in asthma

Airway epithelium serves as the primary physical barrier against exogenous insults. Exposure to allergens, pathogenic microorganisms, and environmental pollutants disrupts epithelial barrier integrity, driving airway epithelial cells (AECs) to secrete epithelium-derived cytokines including thymic stromal lymphopoietin (TSLP), interleukin-25 (IL-25), and interleukin-33 (IL-33). These cytokines potently activate DCs and ILC2s, thereby initiating the downstream immune cascade response (11). ILC2s belong to the innate lymphoid cell family. Unlike T lymphocytes, ILC2s lack the expression of antigen-specific receptors (12). ILC2s orchestrate innate type 2 immune responses. These cells not only initiate airway inflammation but also regulate the recruitment and activation of other innate and adaptive immune cells, further amplifying inflammatory cascades (13). Accumulating evidence indicates that ILC2s-derived IL-13 facilitates dendritic cell migration and potentiates their antigen-presenting capacity (11). More importantly, ILC2s exhibit prominent cytokine plasticity. Under specific microenvironmental cues, ILC2s are capable of producing type 1- and type 17-associated cytokines, implying their pathogenic involvement in non-allergic neutrophilic asthma (13). Furthermore, ILC2s and Th2 cells constitute the central effector cells driving type 2 immune responses and exert synergistic functions. ILC2-derived cytokines further facilitate Th2 cell differentiation and sustain their effector functions (12).

Pulmonary macrophages also serve as central regulators of innate immunity in asthma. These cells mediate the recruitment of eosinophils, neutrophils, monocytes, and other immune cells, which amplify allergic inflammation and initiate T helper cell responses. Macrophages exhibit remarkable plasticity and polarize into proinflammatory classically activated M1 macrophages or anti-inflammatory alternatively activated M2 macrophages contingent on local microenvironmental cues. In allergic asthma, M2 macrophages exacerbate airway inflammation by potentiating Th2 immune responses (14). As professional antigen-presenting cells, DCs are indispensable during allergen sensitization. Upon activation by epithelium-derived signals, DCs migrate to draining lymph nodes to present antigens to T_N_ and drive their differentiation toward the Th2 lineage. Accordingly, DCs and macrophages are recognized as central regulatory hubs governing type 2 immunity in asthma (15).

At the adaptive immune level, aberrant differentiation and dysfunction of CD4^+^ T cell subsets act as central drivers of immunopathology in asthma. The canonical theory attributes asthma to an imbalance between T helper type 1 (Th1) and Th2 responses; nevertheless, this model fails to fully recapitulate the complex pathogenesis of asthma. To date, multiple CD4^+^T cell subsets, including Th1, Th2, Th17, T helper type 9 cell (Th9), follicular helper T cells (Tfh), and regulatory T cells (Tregs), have been demonstrated to participate in asthma initiation and progression via distinct mechanisms (16).

Th2 cells serve as primary effector cells of type 2 inflammation. They secrete type 2 cytokines, including IL-4, IL-5, and IL-13, which trigger eosinophilic infiltration, IgE class switching, goblet cell metaplasia, and AHR. In a substantial proportion of asthmatic patients, chronic airway inflammation is driven by IL-4/IL-5/IL-13-producing Th2 cells and ILC2s (17).

The balance between Th17 and Tregs carries critical implications for immune regulation in asthma. Th17 cells secrete interleukin-17A (IL-17A) and interleukin-17F (IL-17F), acting as central drivers of neutrophilic inflammation (18). Under homeostatic conditions, Tregs preserve pulmonary immune tolerance and tissue homeostasis to restrain excessive inflammatory responses (19). In chronic asthmatic airway inflammation, the regulatory capacity of pulmonary Tregs collapses; Treg cells shift toward a pathogenic phenotype and amplify Th2-, Th17-, and ILC2-mediated airway responses via upregulated Notch4 expression (20). In other words, Treg cells exert dual roles in asthma, serving as both immune modulators and potential disease effector cells. Th17/Treg imbalance is recognized as a pivotal driver of corticosteroid-resistant neutrophilic inflammation in severe asthma (21).

### 2.2 Core molecular pathways underlying asthmatic airway inflammation

Dysregulated functions of the aforementioned immune cells are finely tuned by multiple intracellular signaling cascades. The nuclear factor kappa-B (NF-κB), signal transducer and activator of transcription 3/signal transducer and activator of transcription6 (STAT3/STAT6), and pyroptosis pathways, together with intricate cytokine networks, collectively form the molecular regulatory machinery underlying asthmatic airway inflammation.

The NF-κB cascade acts as a central transcriptional hub sustaining chronic airway inflammation in asthma. Asthma pathophysiology is tightly linked to dysregulation of core intracellular signaling pathways, including NF-κB, Janus kinase-signal transducer and activator of transcription (JAK-STAT), Mitogen-Activated Protein Kinase (MAPK), Phosphatidylinositol 3-kinase (PI3K), and Nuclear factor erythroid 2-related factor 2 (Nrf2). NF-κB potentiates the expression of proinflammatory cytokines in AECs to perpetuate chronic inflammatory responses. Persistent NF-κB activation in asthmatic airways drives macrophages to produce inflammatory mediators such as tumor necrosis factor-α (TNF-α) and interleukin-1β (IL-1β), which further exacerbate inflammation and airway resistance (22). Moreover, the oxidative stress-PARP-1-NF-κB signaling axis is strongly coupled to asthmatic inflammatory responses (23).

The STAT3 and STAT6 signaling cascades exert distinct yet complementary functions in immune polarization during asthma. As the master transcription factor downstream of IL-4 and IL-13, STAT6 occupies a central position in the pathogenesis of allergic disorders. STAT6 transduces IL-4/IL-13-elicited tissue responses that recapitulate hallmark asthmatic pathologies, including eosinophilic inflammation, AHR, subepithelial fibrosis, and excessive mucus secretion (24). Genome-wide and exome sequencing studies have linked gain-of-function STAT6 variants to early-onset, severe and multi-comorbid allergic diseases accompanied by asthma (25). In contrast, activated STAT3 drives Th17 responses and cooperates with NF-κB to trigger pro-survival and anti-apoptotic signaling (26). Preclinical animal studies demonstrate that selective STAT3 inhibition markedly alleviates pulmonary inflammation, highlighting its therapeutic target potential for asthma (27).

Pyroptosis, a pro-inflammatory form of programmed cell death, has been established as a crucial contributor to the pathogenesis of asthma. This process is initiated by the activation of the NLR Family Pyrin Domain Containing 3 (NLRP3) inflammasome, which subsequently triggers caspase-1 activation and promotes the maturation and extracellular release of IL-1β and interleukin-18 (IL-18) (28). In asthmatic airways, diverse stimuli, including environmental allergens, respiratory viral infections, and inhaled pollutants, can induce pyroptosis and concurrently activate its associated signaling cascades. Hyperactivation of the NLRP3 inflammasome exacerbates airway inflammatory cell infiltration, thereby driving AHR and structural remodeling (29). Considerable attention has been directed toward the differential roles of the NLRP3 inflammasome and its downstream effectors IL-1β, IL-18, and IL-33 across distinct asthma endotypes. Furthermore, Gasdermin D (GSDMD)-mediated pyroptosis is emerging as a promising precision anti-inflammatory target for the management of acute and chronic pulmonary diseases (30).

Cytokine networks serve as terminal effector cascades downstream of the aforementioned signaling pathways, orchestrating the regulatory system of asthmatic inflammation. Epithelium-derived cytokines occupy a central position in initiating and sustaining asthmatic inflammation. Upon activation, AECs release alarmins including TSLP, IL-25 and IL-33, which trigger the downstream IL-4/IL-5/IL-13 axis to drive eosinophilia, elevated serum IgE and aggravated AHR. IL-25 acts as a master initiator of type 2 inflammation in T2-high asthma. Beyond inflammatory infiltration, IL-4 and IL-13 induce structural airway remodeling characterized by subepithelial fibrosis, smooth muscle hypertrophy, and goblet cell hyperplasia. Proinflammatory cytokines amplify inflammatory responses by activating immune cells and launching cytokine cascades, whereas chemokines guide the recruitment of immune cells to inflamed airway lesions. These cytokines form positive feedback loops with previously described NF-κB, STAT3, and STAT6 signaling pathways, collectively sustaining and exacerbating chronic airway inflammation in asthma (22, 31).

## 3 Lung microbiome dysbiosis and its crosstalk with host immunity in asthma

### 3.1 Structural and diversity characteristics of lung microbiome in asthma

**Figure 2:**
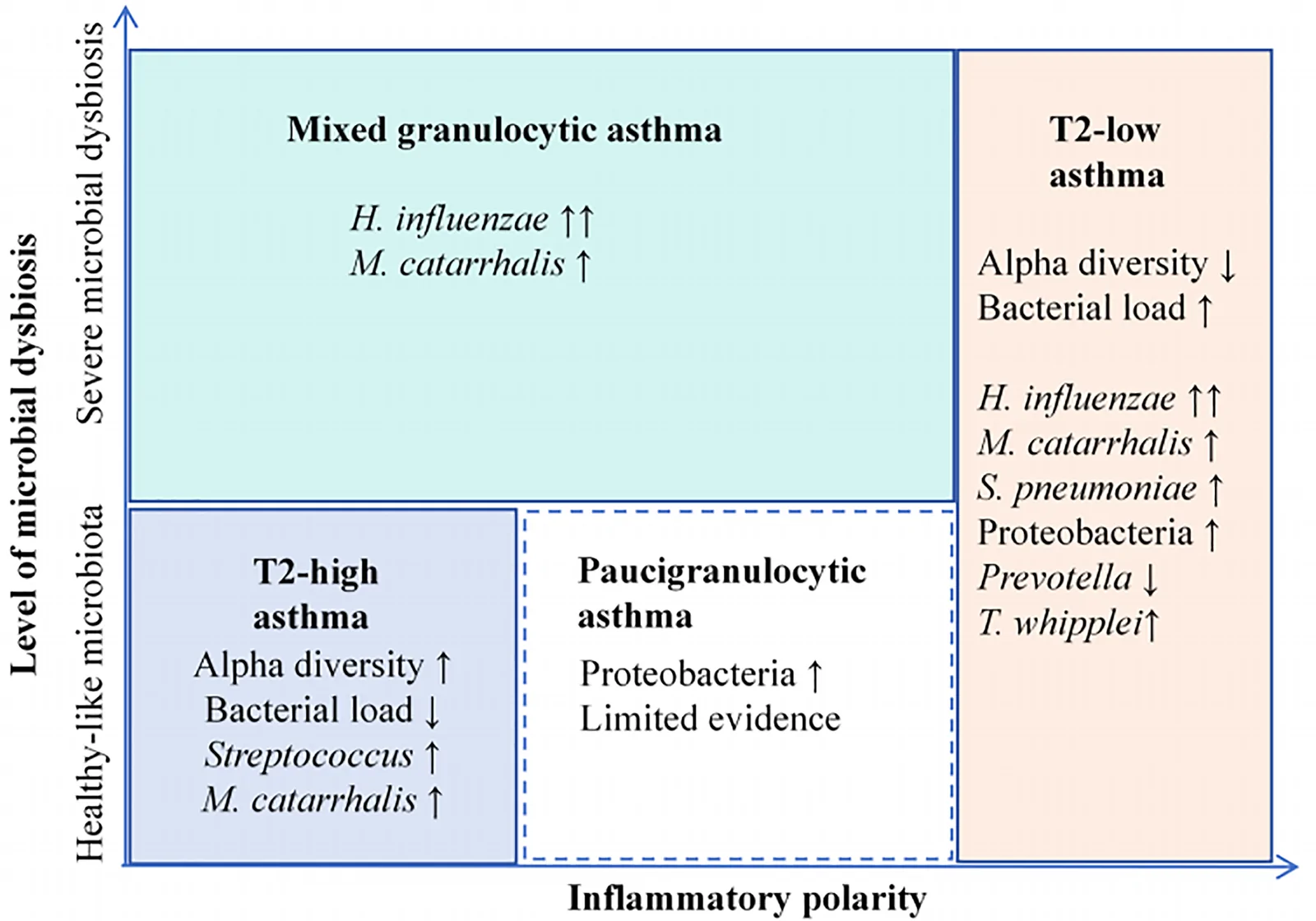
Microbial dysbiosis in four major inflammatory endotypes of asthma: pathogen enrichment and reduced diversity. Across four asthma endotypes (T2-high, T2-low, mixed, and paucigranulocytic), lung bacterial composition varies heterogeneously. T2-high: increased α-diversity; T2-low (neutrophilic): pathogen enrichment and reduced diversity (dysbiosis). Mixed granulocytic (both eosinophil and neutrophil elevation): limited data. Paucigranulocytic (neither elevated): no high-quality data on characteristic genera.

Lung microbiome composition and diversity in asthmatic patients differ across inflammatory phenotypes (**Figure 2**), including eosinophilic, neutrophilic, mixed granulocytic, and paucigranulocytic asthma. Neutrophilic asthma is associated with greater disease severity, longer duration, glucocorticoid resistance, elevated airway neutrophils, and higher healthcare resource utilization.

In T2-low asthma, characterized by neutrophilic infiltration, elevated Interleukin-8 (IL-8), and NLRP3 inflammasome activation, airway microbial diversity is markedly reduced, with significant compositional alterations in the lung microbiome. Notably, *H. influenzae* and *M. catarrhalis* are enriched and become dominant taxa in the lower respiratory tract; their colonization is closely associated with glucocorticoid resistance and exacerbated airway inflammation (32, 33). Neutrophilic asthma accounts for approximately 20% of adult asthma cases. With age-related decline in neutrophil function, particularly microbial killing capacity, airway bacterial load increases, microbial diversity decreases, and Gammaproteobacteria emerge as the dominant taxa, a microbiome profile resembling that seen in other chronic respiratory diseases, including bronchiectasis and chronic obstructive pulmonary disease (COPD) (34). Similarly, in patients with neutrophilic asthma, the relative abundance of *H. influenzae* and *M. catarrhalis* in sputum is significantly increased, whereas in patients with eosinophilic asthma, the abundance of *T. whipplei* is elevated. Patients with severe asthma (including non-smoking, smoking, and former smoking severe asthma) exhibit lower α-diversity, and their lung microbiome remains relatively stable over one year (35). Analysis of the four inflammatory phenotypes via Faith’s phylogenetic diversity revealed that patients with neutrophilic asthma exhibited reduced lung microbial diversity, with significantly increased abundance of *Moraxella* and *Haemophilus*; additionally, the proportion of neutrophils was significantly negatively correlated with α-diversity (36). These observations suggest that specific asthma inflammatory phenotypes are closely associated with lung microbiome dysbiosis, with distinct microbial signatures linked to clinical features across different phenotypes, further supporting an interaction between loss of microbial diversity and neutrophilic airway inflammation.

In T2-high asthma, characterized by IL-4, IL-5, and IL-13 as hallmarks that promote eosinophil recruitment and survival, the lung microbiome exhibits distinct features. Both fungal and bacterial diversity in patients with T2-high asthma are significantly lower than those in T2-low patients. Specific bacterial genera, such as *Stenotrophomonas*, show higher abundance in patients with allergic asthma, and the fungal-bacterial co-occurrence network structure differs from that in healthy controls (37). T2-high asthma typically exhibits lower total airway bacterial load and higher microbial diversity, with significantly lower total bacterial load in induced sputum samples compared to patients with T2-low asthma (38). Such discrepancies indicate divergent host–microbe crosstalk among distinct asthma endotypes. Some studies have shown that there is no significant difference in the lung microbiome composition between asthmatic patients in the high eosinophil group and healthy controls, whereas the low eosinophil group exhibits increased diversity (higher α-diversity and lower β-diversity), with increased abundance of the genera *Neisseria*, *Bacteroides*, and *Rothia*, and decreased abundance of *Sphingomonas*, *Halomonas*, and *Aeribacillus* (39). This correlation reveals that eosinophil abundance may is a key factor governing microbial community structure. Both bacteria of the phylum Firmicutes and *T. whipplei* have also been found to be associated with eosinophilic asthma (40). These findings implicate microbe–microbe interactions in disease pathogenesis. Based on the structure of the lung microbiome, asthmatic patients can be classified into distinct community types (pulmotypes). For instance, pulmotype P1 is dominated by *Pasteurellaceae*, *Streptococcus*, and *Rothia* as dominant taxa, and is associated with higher eosinophil levels; pulmotype P3, on the other hand, is dominated by *Faecalibacterium* and *Bacteroides*, and is associated with increased neutrophils (41). These findings further support a potential role for the microbiota in asthma subtyping.

Studies on the lung microbiome in mixed granulocytic and paucigranulocytic asthma are relatively limited; however, the overall trend indicates that the lung microbiome in asthmatic patients generally exhibits dominant enrichment of Proteobacteria (e.g., *Haemophilus* and *Moraxella*). In contrast, healthy lungs are dominated by Firmicutes, Bacteroidetes, Actinobacteria, and others, while the diversity of Proteobacteria in the lungs of asthmatic patients is increased (42), indicating that the lung microbiome in this form of asthma is characterized by expansion of specific bacterial phyla. The dominant lung microbiome in asthmatic patients also includes *Parabacteroides*, *Leptotrichia*, *Bacteroides*, and others, whereas healthy controls are enriched with *Streptococcus*, *Corynebacterium*, *Porphyromonas*, etc. Among these, the abundance of *Leptotrichia* is positively correlated with the asthma control test (ACT) score, and the abundance of *Phocaeicola* is negatively correlated with airway eosinophils (43). Findings suggest that microbiota composition may reflect disease activity and inflammatory phenotype. Some studies have shown that airway microbial diversity in asthmatic patients is higher than that in healthy controls, and is correlated with the degree of bronchial hyperreactivity, involving families such as *Comamonadaceae* and *Sphingomonadaceae* within Proteobacteria. However, community diversity is not specific to asthma, as some healthy individuals also exhibit high diversity (44). These findings suggest that microbial diversity alone may not serve as a reliable asthma biomarker. Mixed granulocytic and paucigranulocytic asthma remains understudied, and its microbiota profiles and clinical relevance await further elucidation.

Animal models and neonatal studies further support the association between the lung microbiome and asthma inflammatory phenotypes. In house dust mite (HDM) induced asthmatic mice, the abundance of *Staphylococcus* in the lungs is increased. Among them, the CNCM I 4969 strain exerts a protective effect, reducing inflammatory cell infiltration and decreasing the percentage of eosinophils. In contrast, the CNCM I 4970 strain exacerbates inflammation, increasing neutrophils and IgE levels (45). These findings suggest that microbial community effects on disease are mediated by strain-specific functions rather than by overall community composition. In the mouse asthma model, the microbiome undergoes significant changes across stages: *Pseudomonas* exhibits high abundance during the acute inflammatory phase, while *Staphylococcus* and *Cupriavidus* show increased abundance in the airway remodeling phase (46), suggesting that the lung microbiome undergoes dynamic shifts during disease progression and may contribute to the regulation of distinct pathological phases. Intranasal administration of inter-alpha-trypsin inhibitor heavy chain 4 (ITIH4) reshaped lung microbiome composition in ovalbumin (OVA)-challenged asthmatic mice. ITIH4 treatment enriched Gram-positive taxa including Nocardioidaceae and Acholeplasmataceae while depleting Gram-negative Helicobacteraceae, which markedly alleviated OVA-triggered type 2 asthmatic inflammation (47). These findings suggest a role for microbiota-targeted interventions in reshaping microbial composition and alleviating asthma phenotypes. In the chronic feline asthma model, the lower respiratory tract microbiota shifts from being dominated by Pseudomonadaceae (in the healthy state) to being dominated by Coccobacillaceae and Flavobacteriaceae, accompanied by the loss of Pseudomonadaceae and a significant decrease in diversity (48). These findings reveal shared microbiota alterations across asthma models of different species. The asthmatic mouse model with delayed early microbial maturation (DMM) reveals that delayed development of the lung microbiome can exacerbate airway hyperreactivity by promoting Th17 cell responses (49), highlighting the critical role of early life microbial colonization in asthma development.

Clinical studies have demonstrated that neonatal lung microbial colonization exerts a profound impact on asthma development. Colonization with *Streptococcus pneumoniae*, *M. catarrhalis*, or *H. influenzae* in 1-month-old neonates significantly increases the subsequent risk of developing the first wheezing episode, persistent wheezing, acute severe wheezing exacerbations, and wheezing-related hospitalization. By the age of 5 years, the asthma diagnosis rate reaches 33% (compared with 10% in non-colonized infants) (50). Colonization with these pathogenic bacteria is also associated with the risk of early-onset pneumonia and bronchiolitis (51). This also suggests that early life respiratory colonization serves as an important predictor of subsequent pulmonary disease. For example, colonization with *M. catarrhalis* and *H. influenzae* elicits a mixed Th1/Th2/Th17 response, with increased levels of IL-1β, TNF-α, etc. *Staphylococcus aureus* primarily induces a Th17 response (with elevated interleukin-17, IL-17), while *S. pneumoniae* may affect immune regulation through immune evasion (52). This implicates aberrant Th2-biased immune responses to specific airway bacteria during infancy as a key mechanism in the development of subsequent eosinophilic asthma. In severe bronchiolitis in infants, specific microbe-virus co-occurrence profiles (e.g., co-occurrence of respiratory syncytial virus (RSV)/rhinovirus A (RV-A) with *Haemophilus*) are associated with an increased subsequent risk of asthma (53). The mechanisms underlying their interactions, whether synergistic, antagonistic, or sequential, and their collective modulation of the host immune system, remain unclear. Additionally, whether these findings extend to broader populations or to neonates of other ethnicities and geographic regions remains to be determined.

Furthermore, comorbid conditions and treatment responses are also associated with the lung microbiome. Among asthmatic patients, sputum bacterial profiles and fungal richness differed substantially between obese and non-obese individuals. In obese asthmatics, airway taxa such as Prevotella exhibited statistically significant correlations with immune mediators, including IL-1β. The distinct microbe-immune signature observed in obese asthma suggests the potential involvement of lung microbiome that may impact the mechanisms or clinical outcomes of obese asthma (54). Treatment-resistant severe asthma patients often have lung microbiome dominated by a single bacterial species (*M. catarrhalis*, *Haemophilus*, *Streptococcus*), and exhibit a higher proportion of neutrophils in sputum and lower lung function (55). This indicates that microbial dysbiosis represents a key feature of treatment-resistant asthma. In mild atopic asthmatic patients, bacteria such as *Haemophilus* and *Neisseria* are enriched in the bronchial microbiome, and certain taxa, including members of the Lactobacillales, are relatively depleted. And the heterogeneity of microbial community composition is higher than that in atopic non-asthmatic individuals. Functional prediction analysis suggests that amino acid and short-chain fatty acid metabolic pathways may be involved in asthma pathogenesis (56). Bronchoalveolar lavage fluid (BALF) and induced sputum from asthmatic patients are often colonized with Gram-negative bacteria such as *H. influenzae*, *M. catarrhalis*, and *S. pneumoniae*, and these bacteria may have already existed before disease onset (57). These findings implicate early microbial colonization in asthma pathogenesis.

The lung microbiome plays a critical role in asthma pathogenesis, inflammatory phenotypes, and therapeutic responses. Distinct asthma phenotypes correlate with specific microbial compositions; certain taxa are linked to disease activity, corticosteroid resistance, and immune response patterns. Animal models support dynamic microbial shifts and causal contributions during disease progression, and neonatal studies highlight the long-term impact of early-life colonization on asthma risk. However, most evidence is cross-sectional, precluding causal inference. Heterogeneity in sample types, phenotyping, and analytical methods limits comparability. Mechanistic studies largely rely on predictive functional profiling without metagenomic, metabolomic, or longitudinal validation. Certain subtypes (e.g., mixed and paucigranulocytic asthma) remain underexplored, and the generalizability of current findings across diverse populations requires further investigation.

### 3.2 The roles of key immune cells in the lung in asthma and their interactions with microbes

**Figure 3:**
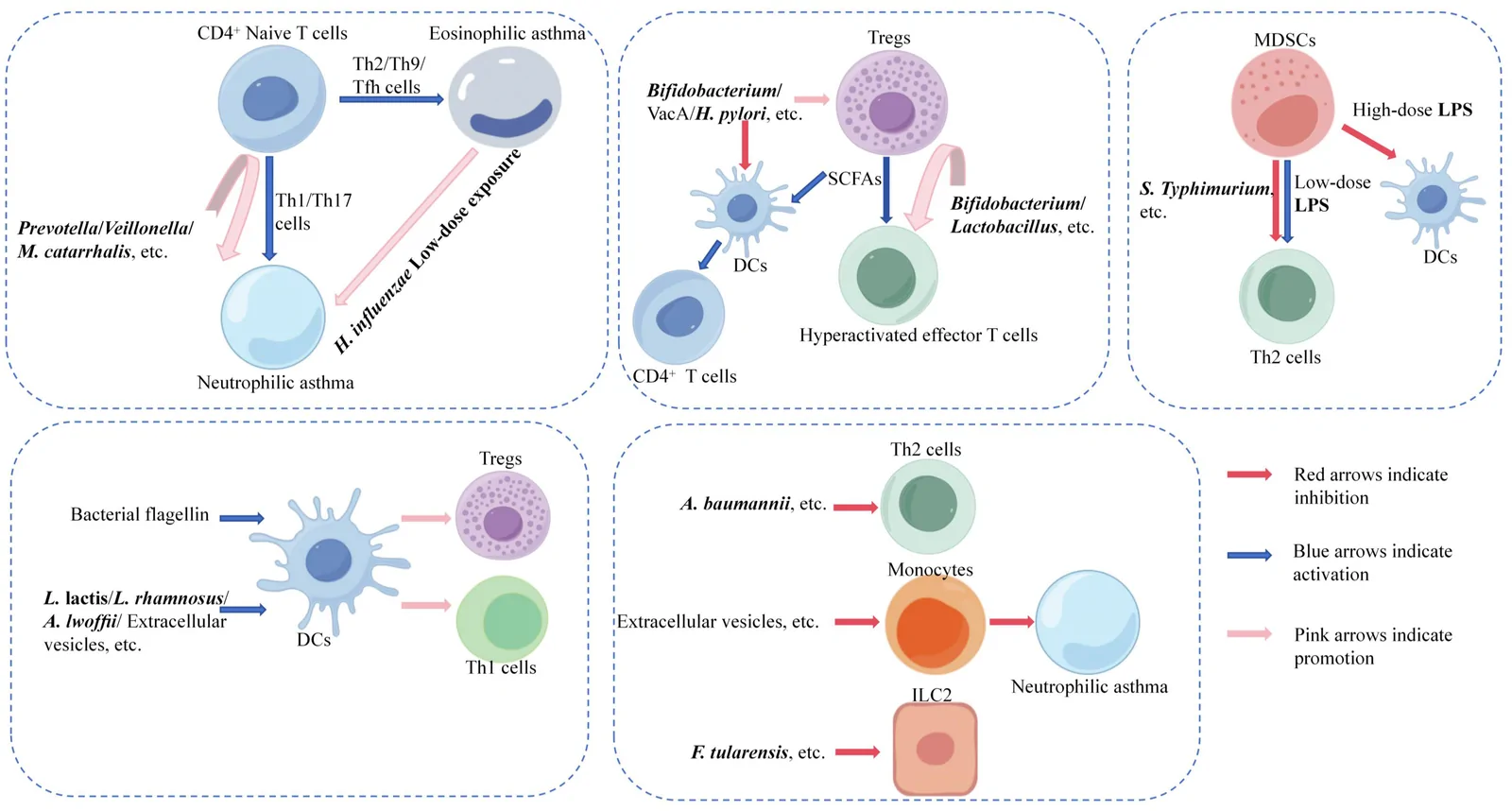
Roles of key pulmonary immune cells in asthma and their crosstalk with the microbiology. Diverse CD4^+^ T-cell subsets dictate asthma inflammatory endotypes: Th2, Th9, and Tfh cells drive eosinophilic asthma, whereas Th1 and Th17 cells are associated with neutrophilic asthma. Tregs restrain excessive effector T cells to maintain immune homeostasis, and myeloid-derived suppressor cells (MDSCs) exert immunosuppressive functions. DCs orchestrate T-cell polarization. Within this network, microorganisms, through their metabolites, structural components, and non-canonical pathways, directly modulate the activity of each immune cell type, thereby shifting the lung immune equilibrium toward or away from asthma pathogenesis.

Multiple subsets of immune cells in the lung exert distinct functions during the development and progression of asthma, whereas microorganisms influence the homeostasis of the immune microenvironment (**Figure 3**). CD4^+^ T cell subsets are key determinants of asthmatic inflammatory phenotypes, among which Th2, Th9, and Tfh mediate the development of eosinophilic asthma, while Th1 and Th17 cells are associated with neutrophilic asthma. Th2 cells secrete IL-4, IL-5, and IL-13, which promote eosinophil infiltration, goblet cell hyperplasia, and IgE production. Their differentiation is regulated by IL-4 secreted by DCs, while ILC2s promote the expression of transcription factors, including GATA3 and STAT6 (58, 59). Th9 cells enhance the function of Th2 cells by secreting interleukin-9 (IL-9), with their transcription factors being PU.1 and Irf4 (60, 61). Tfh cells differentiate in lymph nodes, promote B cell class switching to IgE via IL-4 and IL-21 (62, 63), and may also migrate to the lungs or transdifferentiate into pathogenic Th2 cells (64, 65). In non-Th2 asthma, Th1 cells secrete IFN-γ, whereas Th17 cells secrete IL-17A, IL-17F, and interleukin-22 (IL-22) to promote neutrophil recruitment. Th17 differentiation depends on IL-6, interleukin-23 (IL-23), and transforming growth factor-β (TGF-β), and its transcription factors include STAT3, RORγt, and RORα (66). Lung microbiome influence inflammatory phenotypes by modulating CD4^+^ T cell differentiation. For instance, *Prevotella* and *Veillonella* are associated with Th17-mediated pulmonary inflammation, while *M. catarrhalis* infection can induce CD4^+^ T cells to produce IL-17 and IFN-γ, thereby exacerbating allergic airway disease (67). The oral commensal bacterium *Streptococcus mitis* can induce local Th17 immune responses in the lungs after intranasal immunization, with CD4^+^ T cells producing large amounts of IL-17A (68). Delayed maturation of the gut microbiota in early life exacerbates Th17-driven asthma with long-term consequences. IL-17A and IL-13 synergistically enhance inflammatory gene expression in pulmonary mesenchymal cells, while anti-IL-17A antibodies can reverse AHR (49). Long-term low-dose exposure to *H. influenzae* shifts Th2-type eosinophilic inflammation to Th17-associated neutrophilic inflammation, accompanied by upregulation of IL-17 and chemokine C-X-C motif ligand 1 (CXCL1) and impaired Treg function (33, 69).

Tregs maintain immune homeostasis by suppressing excessive and pathogenic effector T cell responses. Probiotics such as *Lactobacillus* and *Bifidobacterium* can promote the differentiation of Tregs and the production of short-chain fatty acids (SCFAs), while inhibiting Th2 and Th17 responses (70, 71). Intratracheal administration of OM-85 increases the number of FOXP3^+^ CD3^+^ CD4^+^ Tregs in the lungs and their activation markers (CTLA-4, CD69), elevates the ratio of Tregs to IL-13^+^ Th2 cells, and drives the immune microenvironment toward tolerance (72). These findings suggest that Probiotics and microbial derivatives suppress asthma inflammation by promoting Treg differentiation. SCFAs, notably butyrate and propionate, epigenetically enhance Treg function by inhibiting histone deacetylases (HDAC) activity in DCs, which reduces inflammatory cytokine expression and promotes Treg differentiation, while also directly acting on CD4^+^ T cells to augment Foxp3 acetylation and stability (73). The lung microbiome of neonatal mice can induce the generation of Tregs to promote allergen tolerance, whereas antibiotic use, resulting in reduced lung microbiome diversity, decreases the number of Tregs, increases IgE levels, and elevates the risk of asthma (74). Indicating that the early-life lung microbiome is critical for Tregs induction and allergen tolerance. The VacA virulence factor of *Helicobacter pylori* promotes the differentiation of Tregs by inducing tolerogenic DCs, and depletion of Tregs reverses its anti-asthmatic effect (75). *H. pylori* infection can also increase the number of Tregs in the lungs, and transplantation of purified Tregs can confer an asthma-protective effect (76). These findings further demonstrate that specific bacterial strains and pathogen virulence factors confer protective effects against asthma.

MDSCs exert immunosuppressive effects in asthma. The bacterial product LPS promotes Th2 responses at low doses, while at high doses, it induces CD11b^+^Gr1(int)F4/80^+^ cells (with a phenotype similar to MDSCs) in lung tissue via the TLR4/MyD88 pathway. These cells inhibit the ability of DCs to promote GATA-3 expression and STAT5 activation in Th2 cells, thereby suppressing airway inflammation (77). Furthermore, MDSC function depends on specific signaling pathways and migratory properties; these cells lack CCR7 and do not migrate to draining lymph nodes. Instead, they inhibit the reactivation of sensitized Th2 cells and the secretion of IL-5 and IL-13 by producing interleukin-10 (IL-10) and arginase 1 (Arg1). Notably, this inhibitory effect is abrogated in MyD88-deficient mice (78). Pathogen infection can also induce MDSCs that confer protection against asthma. CD11b^+^Gr1^+^ myeloid cells induced by *Salmonella Typhimurium* infection also exert an inhibitory effect by downregulating GATA-3 expression and IL-4 production in Th2 cells, and this mechanism depends on the activity of inducible nitric oxide synthase (iNOS) and arginase (79). This underscores the complexity of post-infection immune modulation.

DCs, as antigen-presenting cells, influence asthmatic phenotypes through Treg differentiation. Pulmonary DCs promote Th2 cell differentiation through antigen presentation and costimulatory signaling, while IL-4, mainly produced by basophils, mast cells, ILC2s, and Th2 cells, further amplifies the Th2 response and drives asthmatic phenotypes. Specific microbes and probiotics confer anti-asthmatic protection by modulating Th1/Treg responses via DCs. Bacterial flagellin induces regulatory DCs, characterized by high expression of IL-10 and indoleamine 2,3-dioxygenase (IDO), by activating toll-like receptor 5 (TLR5) on DCs. This process promotes the generation of Foxp3^+^ Tregs and suppresses airway inflammation induced by OVA or HDM (80). After being taken up by DCs, *Lactococcus lactis* G121 relies on endosomal acidification to activate toll-like receptor 13 (TLR13) (in mice) or toll-like receptor 8 (TLR8) (in humans) for bacterial RNA recognition. This process induces DCs to secrete IL-12 and IL-10, which promote the polarization of CD4^+^ T cells toward the Th1 subtype and reduce eosinophil infiltration (81). The probiotic *Lactobacillus rhamnosus* aerosol promotes the maturation of CD103^+^ and CD11b^+^ DCs in the lungs, with upregulation of CD80, CD86, and MHC class II (82). *Acinetobacter lwoffii* F78 and *L. lactis* promote Th1-biased immune responses and alleviate allergic inflammation by activating costimulatory molecules on the surface of DCs and secreting IL-12 (83). Extracts of *H. pylori* induce BATF3-dependent CD103^+^CD11b^-^ DCs to secrete IL-10 via the TLR2/MyD88 pathway, and this DC subset is indispensable for asthma protection (84). Furthermore, the lung microbiome promotes immune tolerance to allergens in neonates by regulating PD-L1 expression on DCs, while the metabolite 12,13-diHOME reduces the number of Tregs by affecting the function of DCs, thereby exacerbating pulmonary inflammation (74, 85).

The lung microbiome directly regulates immune cell functions through diverse and non-canonical mechanisms. For instance, pulmonary infection with *Acinetobacter baumannii* directly inhibits local Th2 cytokine expression in the airways and reduces eosinophil infiltration, and this effect occurs independently of the TLR4 and IFN-γ pathways (86). Extracellular vesicles derived from *L. lactis* activate DCs to produce IL-12, thereby promoting Th1 differentiation and IFN-γ secretion and suppressing Th2 inflammation (87). These findings expand current knowledge of microbe-immune crosstalk. High-abundance colonization of early nasopharyngeal microbiota (e.g., *Streptococcus*) is associated with asthma risk, and may indirectly regulate asthma development by influencing the immune cell environment (88). These findings further imply that early-life colonization by specific microbiota influences later asthma risk via modulation of the local immune environment. Bacterial exosomes derived from *M. luteus* inhibit IL-1β production by monocytes and the activation of ILC3s, reduce neutrophil recruitment, and alleviate neutrophilic airway inflammation (89). This offers potential therapeutic avenues for refractory neutrophilic asthma, suggesting that bacterial extracellular vesicles hold potential for modulating innate immunity and neutrophilic inflammation. Bacterial infections and their products significantly affect the numbers and functions of ILC2s, thereby regulating asthma pathological processes. During infection with bacteria such as *Francisella tularensis* LVS, specific cytokine IFN-γ can be induced in the inflammatory milieu, which inhibits GATA3, a key transcription factor of ILC2s, via the JAK-STAT1 pathway, blocks their survival signals, and thereby leads to a significant reduction in the number of pulmonary ILC2s. Furthermore, IL-5 produced by ILC2s may play a role in limiting protective immunity following infection, and blockade mediated by anti-IL-5 antibodies can reduce bacterial burden after infection (90–92). These findings highlight the complex bidirectional crosstalk of ILC2s in infection and asthma.

### 3.3 Molecular mechanisms of host immune regulation by microbial components and metabolites

Microbial products bidirectionally regulate immune cell activity through a four-tiered cascade: PAMP-PRR recognition, intracellular signaling, target cell responses, and inflammatory mediator output, ultimately shaping either a pro-inflammatory or tolerogenic immune microenvironment (**Table 1**). Microbial products regulate immune cell activity and the expression of inflammatory mediators through multiple molecular mechanisms, among which LPS, exosomes, bacterial lysates, and flagellin play important roles. As a component of the Gram-negative bacterial cell wall, LPS can trigger signaling pathways via PRRs, such as TLR4. For instance, in LPS-induced asthmatic phenotypes, IL-12 production is influenced by the IL-13Rα2 restrictive receptor, which in turn regulates the IL-12-STAT4-IFN-γ axis to modulate immune responses (103). After forming a complex with the accessory factor myeloid differentiation protein-2 (MD2) to recognize LPS, TLR4 activates MyD88-dependent and TRIF-dependent pathways, which induce the expression of proinflammatory cytokines and type I interferons via NF-κB and IRF3, respectively (104). Furthermore, bacterial extracellular vesicles (BEVs), which are microbial-derived membrane vesicles, carry MAMPs, including LPS. They enter host cells through pathways such as membrane fusion and endocytosis, activate receptors like TLR4, induce proinflammatory factors, TNF-α, and IL-6 in lung ECs, and recruit neutrophils and macrophages, thereby participating in pulmonary inflammation in diseases such as asthma. The diversity of microbial product–receptor signaling axes provides potential therapeutic targets for asthma intervention.

**Table 1:** Molecular mechanisms underlying microbial product regulation of immune cell activity and inflammatory mediator expression.

Microbial product	Receptor recognition	downstream signaling	Target immune cells	Inflammatory output	Reference
LPS	TLR4 · MD-2 · CD14 (Cell membrane localization and recognition)	MyD88/NF-κB/TRIF/IRF3·MAPK	Macrophages, DCs, MCs, AECs, ILC3s	IL-1β, TNF-α, IL-6, IL-33 Th2 cytokines IL-17 (Neutrophilic inflammation)	(93, 94, 45)
OMVs/BEVs/EVs	TLR2 · TLR4 · TLR9 (Surface PAMPs and nucleic acids)	NF-κB · MAPK	Macrophages, DCs, Alveolar epithelial cells	IL-1β, TNF-α, IL-6, IFN-γ Antigen-specific antibodies Anti-inflammatory activity (probiotic EVs)	(95, 96)
Bacterial lysate (eg. OM-85)	Multi-TLR agonist (TLR2/4/7/8/9)	MyD88/Trif-dependent/Tolerogenic DC reprogramming	DCs, Epithelial cells, ILC2s, T cells (Treg induction)	IL-33↓ · Th2 Cytokines↓ IL-10↑ · TGF-β↑ Eosinophilic inflammation ↓	(72)
Flagelin	TLR5 (Plasma membrane) NLRC4 inflammasome (Cytosolic)	MyD88/NF-κB Inflammasome/Caspase-1	AECs, DCs Macrophages, T cells	Pro-inflammatory: IL-1β, TNF-α, IL-6, IL-8 Immunomodulation: Induction of rDCs/Tregs (low dose) Th2/Th17 polarization (adjuvant effect)	(80, 97)
Bacterial DNA (CpG)	TLR9 (Endosomal)	MyD88/NF-κB · AP-1	plasmacytoid DCs (pDCs), B lymphocytes, Macrophages	IL-12, IL-8, IFN-α/β Th1 polarization, antibody response	(93, 98)
Petidoglycan	TLR2 (Plasma membrane) NOD2 (Cytosolic)	TLR2/NF-κB NOD2/RIP2/NF-κB	Macrophages, DCs Keratinocytes	IL-10, TNF-α Th17 polarization (NOD2-dependent)	(99–101)
SCFAs (Butyrate,etc.)	GPCR (GPCR41/43/109A) HDAC inhibitors (epigenetic)	GPCR/MAPK · PI3K HDAC inhibition/Foxp3 ↑	T cells, DCs, MDSCs	Tregs ↑ · IL-10 ↑ Th2/Th17 cytokines ↓ IgE ↓ · Anti-inflammatory effects	(73, 102)

↑indicates upregulation, while ↓ represents downregulation.

Exosomes and metabolites of microbial origin exhibit dual immunomodulatory functions. Extracellular vesicles (EVs) derived from *Clostridium butyricum* inhibit the TLR4/MyD88/NF-κB signaling pathway, thereby reducing the expression of proinflammatory cytokines (IL-6, IL-1β, and TNF-α) while upregulating the anti-apoptotic protein Bcl-2 and tight junction proteins (ZO-1 and Occludin). This process protects the function of the pulmonary mucosal barrier and alleviates inflammatory responses in acute lung injury (105). Products of probiotics (e.g., *Lactobacillus* and *Bifidobacterium*), such as bacteriocins and organic acids, can bind to PRRs via MAMPs, including lipoteichoic acid (LTA) and cell wall polysaccharides (CPS), thereby activating Tregs to secrete IL-10. This process balances Th1/Th2 responses and promotes immune homeostasis (106). MAMPs carried by BEVs can also activate the Syk-CARD9 or NF-κB pathways via receptors such as TLR2 and nucleotide-binding oligomerization domain (NOD) receptors. This process induces the expression of TNF-α, IL-6, and chemokines (CXCL1 and CCL2) in lung ECs, thereby recruiting inflammatory cells and exacerbating lung inflammation (107). Thus, microbial products regulate inflammation in a source and receptor-specific manner.

Bacterial lysates, as an important component of microbial products, OM-85, can significantly inhibit eosinophil infiltration and the expression of type 2 cytokines, IL-5 and IL-13, in asthma models through intranasal administration. Its mechanism may involve enhancing airway epithelial barrier function and downregulating inflammatory chemokines *Ccl11* and *Ccl24*, thereby regulating immune cell activity (108). Nevertheless, its role in mixed granulocytic or neutrophilic asthma remains to be further elucidated. Intranasal pretreatment with *Lactobacillus* also exhibits similar immunomodulatory effects; it reduces proinflammatory mediators such as CXCL1 and IL-1β, increases the anti-inflammatory factor IL-10, and decreases neutrophil recruitment. Meanwhile, it activates the phagocytic and bactericidal functions of macrophages, thereby initiating pulmonary immune defense (109). These findings suggest that intranasal pretreatment with specific probiotics, such as *Lactobacillus*, exerts broad-spectrum anti-inflammatory and immunoprotective effects.

Flagellin, a component of the bacterial motility apparatus, can be recognized by TLR5. In intestinal innate immunity, it induces IgA production and Th1/Th17 differentiation, directly regulating immune cell activity. Furthermore, after PRRs, such as NOD-like receptors (NLRs), recognize bacterial peptidoglycan derivatives, they activate NF-κB and inflammasomes, promoting the maturation and release of IL-1β to further participate in inflammatory regulation (110). These mechanisms have been primarily defined in intestinal immunity and await systematic validation in pulmonary DCs, ECs, and asthma-relevant immune cells. Moreover, despite the immunomodulatory potential of flagellin and NLR ligands, their therapeutic window, safety, and efficacy in non-T2 asthma models remain unexplored. Notably, cigarette smoking can lead to neutrophilic inflammation in the airways of asthmatic patients, which is characterized by a non-T2 endotype and is associated with steroid-refractory asthma. While this process does not directly depend on microbial products, it may have crosstalk with microbially regulated immune pathways (111). Whether altering lung microbiome composition indirectly affects lung inflammatory phenotypes remains to be established, and such a link would suggest potential crosstalk between environmental factors and the microbiota–immune axis.

### 3.4 The association between the lung microbiome and clinical phenotypes of asthma

The composition of the lung microbiome is closely associated with asthma inflammatory phenotypes, disease severity, and drug responses. It influences disease phenotypes through multiple mechanisms, including regulation of host immune responses, inflammatory pathways, and pathogen interactions.

Regarding inflammatory phenotypes, early colonization patterns of the lung microbiome and changes in the abundance of specific microbial taxa are important regulatory factors. The succession process of the respiratory microbiota during the neonatal period is closely associated with the subsequent risk of developing asthma and inflammatory severity. A high abundance of commensal bacteria, such as *Corynebacterium* and *Dolosigranulum*, is correlated with reduced rates of respiratory infections and a lower risk of asthma, whereas a microbiota dominated by potential pathogenic bacteria (e.g., *H. influenzae* and *Streptococcus*) is associated with greater infection severity and higher asthma incidence. Additionally, the coexistence of respiratory viruses (e.g., RSV) with these bacteria can enhance inflammatory responses (112, 113). These findings suggest that early microbial composition shapes disease trajectory and that microbial interactions exert synergistic effects in asthma pathogenesis. Molecular products of specific pathogenic bacteria can directly regulate inflammation. For example, outer membrane vesicles (OMVs) from the human respiratory pathogen *M. catarrhalis* carry adhesins (e.g., UspA1/A2) and virulence factors. These OMVs enter alveolar epithelial cells (ECs) through lipid raft-mediated uptake and subsequently activate TLR2 (TLR2) signaling, and those carrying UspA1 inhibit EC inflammatory responses (114). The abundance of *Akkermansia muciniphila* correlates with eosinophilic inflammation. In obese asthmatic patients, its abundance is significantly reduced and negatively correlates with serum C-reactive protein (CRP) levels. Oral administration of *A. muciniphila* alleviated OVA- and HDM-induced airway inflammation in mice, as evidenced by reduced BALF eosinophil counts and decreased lung IL-4 and IL-5 levels (115). These findings also suggest that certain host-commensal bacteria exert anti-inflammatory and protective effects, indicating their potential therapeutic value. Specific pathogens promote eosinophilic inflammation via distinct immune pathways. For example, *S. aureus* promotes eosinophilic inflammation through protease-activated receptor-2 (PAR-2) activation: its secreted serine protease-like proteins (Spls) activate PAR-2 on lung epithelial cells, triggering alarmins (IL-33, TSLP), which activate ILC2s and drive Th2 responses. Notably, *S. aureus* colonization is 87% in non-allergic asthma versus 20% in healthy adults, and closely associates with the eosinophilic inflammatory phenotype (116). *S. pneumoniae* is also associated with eosinophilic inflammation. It affects eosinophil recruitment and Th17 cell differentiation by inducing lymphocyte activation and modulating CD69 expression, thereby promoting Th2-high eosinophilic asthma (41). These findings further support a key role for specific pathogenic bacteria in asthma phenotype differentiation.

Neutrophilic asthma, a refractory inflammatory phenotype, is closely associated with distinct lung microbiome and immune pathways. In adult patients, airway abundance of Proteobacteria (e.g., *Haemophilus*, *Moraxella*) is increased, whereas *Veillonella* and *Prevotella* are decreased. Colonization with *H. influenzae* and *M. catarrhalis* induces a mixed Th1/Th2/Th17 response, while *S. aureus* drives a Th17-specific phenotype. Affecting approximately 10% of asthmatics, this subtype is characterized by elevated airway IL-17, prominent neutrophilic infiltration, and steroid resistance (117). Lung microbiome LPS regulates inflammatory phenotype switching. LPS-enriched OVA challenge induces mixed Th1/Th2/innate responses via the Toll-like receptor 4 (TLR4), driving concurrent eosinophil and neutrophil infiltration. Conversely, TLR4-deficient mice lack LPS-induced neutrophilia but retain partial eosinophilic inflammation (118). In OVA-induced asthma models, LPS exposure shifts inflammation from eosinophil- to neutrophil-dominant, increasing IL-17A and decreasing Th2 cytokines (IL-4, IL-5). This shift is IL-17A-dependent, as IL-17A-deficient mice maintain eosinophilic inflammation (119). LPS at different concentrations can also regulate immune responses via distinct signaling pathways: low-dose LPS promotes TNF-α receptor-mediated Th2 responses, whereas high-dose LPS drives STAT4-dependent Th1 responses, leading to eosinophilic or neutrophilic phenotypes (120). Additionally, specific pathogens directly disrupt the airway barrier and regulate inflammation. For instance, exoproteins from planktonic *Pseudomonas aeruginosa* impair nasal epithelial barrier integrity, reduce transepithelial resistance and disrupt tight junctions, and induce interleukin-6 (IL-6) release. These effects are more pronounced in patients with chronic rhinosinusitis and comorbid asthma, suggesting a role for this bacterium in modulating inflammatory phenotypes (121). These findings suggest that specific microbiota and their components are associated with the inflammatory phenotype of refractory asthma, potentially by modulating it through barrier disruption.

Lung microbiome composition is associated with asthma severity, including onset risk, exacerbation frequency, and disease progression. Following infant hospitalization for bronchopneumonia, transient nasal microbiota changes may predict long-term wheezing. Specifically, increased relative abundance of *Moraxella* and *Streptococcus* at 3 weeks post-hospitalization (clearance phase) is significantly associated with recurrent wheezing before age 3, with risk ratios of 1.38 and 1.76, respectively (122). This study demonstrates that post-infection microbiota recovery trajectories predict subsequent airway health. Neonatal upper respiratory colonization by *H. influenzae* or *M. catarrhalis* was associated with significantly elevated eosinophil and total IgE levels at age 5, correlating with increased risks of childhood asthma and exacerbation (123). Asthmatic patients with specific IgE positivity to *S. aureus* enterotoxins (SEs) usually have more severe disease, which is characterized by decreased lung function, the need for more inhaled or oral steroid therapy, and elevated serum eosinophil cationic protein (ECP) levels (124). Further evidence indicates that neonatal colonization by specific pathogenic bacteria correlates with elevated asthma-related immune markers and exacerbation risk. In severe versus mild-to-moderate asthma, induced sputum shows higher neutrophil proportion and Interferon gamma (IFN-γ) mRNA expression, with neutrophil proportion positively correlating with clinical severity (125, 126). These findings further underscore the central role of neutrophilic inflammation in refractory asthma.

Furthermore, the lung microbiome also regulates drug responses and disease courses in asthma by altering susceptibility to pathogenic infections. Among 407 patients with asthma exacerbations, viral and/or bacterial infections were detected in 50.4%. The infection rate of *Chlamydia pneumoniae* in non-obese patients was 7.6%, which was significantly higher than the 1.0% in obese patients. Moreover, *C. pneumoniae* infection in the non-obese group was associated with a prolonged duration of systemic corticosteroid use (13.6 ± 19.8 days vs. 9.7 ± 6.7 days) and a prolonged duration of exacerbations. In contrast, bacterial infection had no significant effect on treatment efficacy in the obese group (127). Long-term airway colonization with *H. influenzae* can shift steroid-sensitive Th2-type eosinophilic inflammation to steroid-resistant Th17-driven neutrophilic inflammation, accompanied by increased expression of IL-17 and RORγt. In contrast, patients with LPS-induced neutrophilic asthma usually exhibit poor responses to glucocorticoid therapy, suggesting that microbially regulated inflammatory phenotypes are key factors influencing drug responses (117).

Abnormal activation of signaling pathways induced by microbial dysbiosis is closely associated with inflammatory phenotypes. Dysregulated microbiota drives antigen-presenting cells to secrete IL-23, which persistently activates RORγt^+^ Group 3 innate lymphoid cells (ILC3s) and promotes robust systemic release of IL-17 into circulation, and IL-17 signaling activates airway smooth muscle cells via the IL-17RA and IL-17RC, promotes fibrotic deposition and enhanced smooth muscle contractility, and thereby leads to airway remodeling (128, 129). Additionally, the NF-κB signaling pathway plays a crucial role in inflammation amplification; for instance, *Corynebacterium striatum* infection facilitates Th17 cell differentiation to induce abundant IL-17 production, thereby triggering neutrophilic inflammation. Meanwhile, this pathogen activates NF-κB signaling to drastically amplify inflammatory responses. The synergistic effect of these two pathways ultimately initiates and sustains the progression of neutrophilic asthma (130). Neutrophil Extracellular Traps (NETs) induce sustained secretion of chemokines by AECs through activating the TLR4/NF-κB signaling pathway, thereby forming a vicious cycle of neutrophil recruitment - inflammatory mediator release - further recruitment (131). Notably, steroid resistance in neutrophilic asthma is closely associated with microbial dysbiosis and abnormal immune pathways. Studies have shown that glucocorticoids not only fail to effectively inhibit the production of Th17 cell-associated cytokines but may even promote Th17 cell differentiation. Furthermore, stress-induced glucocorticoids increase the production of IL-17 and neutrophil recruitment in response to bacterial antigen stimulation (132). HDM-challenged mice exhibited severe intestinal dysbiosis, marked by depleted Lactobacillus and Bifidobacterium alongside reduced tryptophan metabolites. Gut dysbiosis suppressed the AhR-NRF2 axis, and resultant signaling dysfunction drove type 2 airway inflammation. Exogenous tryptophan remodeled gut microbiota, restored AhR-NRF2 signaling, and alleviated asthmatic phenotypes. Collectively, these data provide direct evidence that gut microbial dysbiosis governs inflammatory asthmatic phenotypes through perturbing canonical AhR-NRF2 signaling (133).

## 4 Asthma treatment strategies and prospects based on the lung microbiome

### 4.1 Advances in research on immunomodulatory therapy targeting microorganisms and their products

In lung microbiome-based immunomodulatory therapy for asthma, bacterial lysates have emerged as a promising strategy, demonstrating significant potential to rapidly induce broad-spectrum anti-infective immunity by activating innate immune pathways. Polyvalent Bacterial Lysates (PBL), such as polyvalent alkaline bacterial lysate (PABL) and polyvalent mechanical bacterial lysate (PMBL), can activate NF-κB via the MyD88-dependent pathway. In lethal *S. pneumoniae* mouse models, they significantly improve survival rates, with the protective effect lasting for 48 to 72 hours. The key protective factors are likely to be small peptide fragments or non-protein components (134). Preclinical and clinical studies indicate that bacterial lysates can potentially reduce inflammation and exacerbation frequency in asthma. In a clinical trial, PMBL tablets reduced the frequency of asthma exacerbations in children aged 6–16 years with partially controlled or uncontrolled allergic asthma by 63% versus placebo at week 12 and by 42% over the entire study period. PMBL^®^ also prolonged the time to the second and third exacerbations and had a favorable safety profile (135). For NTHi-associated neutrophilic asthma, orally administered inactivated NTHi can enhance intestinal antigen delivery, improve the phagocytic function of airway macrophages, and reduce NTHi load in the airways, thus demonstrating therapeutic potential (33). Furthermore, whole-cell lysates of *H. pylori* effectively prevent allergic asthma in mice by inducing the production of IL-10 and IL-18 in DCs, via the CD103^+^ CD11b^-^ dendritic cell subset dependent on the BATF3 transcription factor. Wherein γ-glutamyl transferase (GGT) and the vacuolating toxin VacA are key determinants (84). Owing to the compositional complexity of bacterial lysates, the specific molecules (e.g., lipoproteins, DNA, peptidoglycan fragments, or small peptides) driving particular signaling pathways remain undefined, and the reliance of animal models on acute infection or allergen challenge, rather than the chronic course of human asthma, further hampers clinical translation.

Probiotics and their formulations exert effects in asthma treatment by regulating immune responses. Prophylactic intranasal administration of *L. rhamnosus* GG (LGG) prevented airway inflammation and AHR in a mouse model of birch pollen-induced allergic asthma, reduced eosinophil counts in BALF, and decreased lung Th2 cytokines, such as IL-5 and IL-13. Its efficacy may be attributed to strong adhesion to upper respiratory and intestinal mucosal surfaces (136). In a dust mite-induced asthma model, intranasal inoculation with CNCM I 4969 (isolated from neonatal mouse lungs) reduced total BALF cell count and eosinophil percentage, and alleviated lung inflammation and airway epithelial hypertrophy. In contrast, CNCM I 4970 exacerbated asthmatic features, whereas co-inoculation resulted in an intermediate effect (45). Inhalable dry aerosol formulations of viable lactobacilli (live biotherapeutic product, LBP) reduced neutrophil infiltration in models of bronchopulmonary dysplasia (BPD) and COPD, decreased pro-inflammatory factors, such as matrix metalloproteinase-9 (MMP-9), neutrophil elastase, CRP, IL-6, and IL-8, improved lung structure and function. One key bioactive component, L(+)-lactic acid, inhibited MMP-9 expression (137). Oral administration of the *B. breve* MRx0004 strain exhibits both prophylactic and therapeutic effects in a mouse model of severe asthma. It inhibited neutrophil and eosinophil infiltration in BALF, alleviated peribronchial and perivascular immunopathology, reduced lung pro-inflammatory factors (IL-1α, IL-1β, CXCL2), and increased Tregs (71). The *Staphylococcus sciuri* W620 strain, isolated from farm dust, activates TLR2 and nucleotide-binding oligomerization domain 2 (NOD2) in human monocyte-derived dendritic cells (moDCs). This activation induces moDC maturation and secretion of IL-23, thereby promoting the secretion of IFN-γ. Prophylactic intranasal instillation of this strain significantly inhibits airway inflammation, mucous cell hyperplasia, and AHR in two mouse asthma models induced by OVA and HDM, respectively (138). Most evidence on probiotic therapy comes from mouse asthma models, with no randomized controlled trials confirming efficacy and safety in patients. Probiotic functionality is highly strain-specific, necessitating precise selection, yet no universal guidance exists. Administration routes, dosage, and treatment duration remain unstandardized. The molecular effectors (e.g., surface proteins, metabolites) and their interactions with the host genome are poorly understood, and long-term safety is unknown. These limitations hinder the clinical application of probiotics such as *Lactobacillus* in asthma management.

Microbial exosomes, as novel immunomodulatory mediators, have emerged as promising candidates in asthma treatment. EVs secreted by *L. lactis* are spherical in shape, with a diameter of approximately 60–100 nm, and enriched in proteins such as pyrophosphate kinase. These exosomes can stimulate DCs to produce IL-12, thereby shifting the immune response of asthmatic mice from a Th2-type to a Th1-type profile. They significantly reduce AHR, eosinophil numbers, the secretion of Th2 cell-associated cytokines (e.g., IL-5, IL-13), and mucus production, while exhibiting favorable *in vivo* safety (87). However, the lack of standardized protocols for the isolation, purification, and characterization of exosomes leads to variability in purity, yield, and bioactivity, which may compromise reproducibility. Moreover, the underlying mechanisms remain poorly understood, posing challenges for further practical application. Bacterial extracellular vesicles derived from *M. luteus* (MlEVs) can significantly reduce the number of neutrophils and the levels of IL-1β and IL-17 in BALF when administered intranasally in a mouse model of neutrophilic asthma. They also decrease the number of ILC3s and the expression of transcription factors such as NLRP3, T-bet, and RORγt in lung tissue. The underlying mechanism is associated with the upregulation of hsa-miR-4517 expression in AECs, thereby inhibiting IL-1β production in monocytes. Additionally, clinical studies have shown decreased levels of MlEV-specific IgG4 antibodies in the serum of asthmatic patients, with even lower levels in patients with neutrophilic asthma; these levels are positively correlated with lung function (forced expiratory volume in 1 second percentage, FEV1%) (89). These findings confirm the potential of bacteria-derived exosomes in the diagnosis and treatment of asthma. However, evidence regarding their efficacy across different asthma subtypes, long-term safety, and clinical intervention remains insufficient. Further elucidation of their mechanisms of action and the conduct of well-designed clinical trials are essential steps for clinical translation.

Specific bacterial proteins play a key role in asthma immunomodulation by regulating immune cell function. Bacterial flagellin B (FlaB) suppresses allergic asthma by inducing regulatory dendritic cells (rDCs) and Tregs. In OVA-induced asthma models, intranasal administration of the TLR5 ligand FlaB together with OVA induced rDCs, enhancing IL-10 and TGF-β secretion while reducing pro-inflammatory cytokines, such as IL-12 and TNF-α. This inhibited OVA-specific CD4+ T cell proliferation and increased Foxp3^+^CD4^+^CD25^+^ Treg numbers and their effector molecules. Notably, Treg depletion abrogated the therapeutic effect (80). In acute and therapeutic HDM-induced allergic airway disease models, *H. pylori* VacA reduced airway inflammation by inducing tolerogenic DCs and promoting Tn differentiation into Tregs, thereby alleviating AHR, airway inflammation, and goblet cell metaplasia. It also increased HDM-specific IgG2b and decreased the IgG1/IgG2b ratio (75). Furthermore, after the bacterial RNA of *L. lactis* G121 is recognized by endosomal TLR13 (in mice) or TLR8 (in humans) in DCs, it can induce DCs to produce Th1-polarizing cytokines such as IL-12p70. This further promotes Th1 cell differentiation and IFN-γ production, thereby effectively protecting mice against OVA-induced asthma-like airway inflammation; this process depends on endosomal acidification and phagocytosis (81). *H. pylori* infection induces increased numbers of pulmonary Tregs (CD4^+^ FoxP3^+^ Tregs) and inhibits dendritic cell maturation in mice, thereby preventing asthma induced by OVA and HDM. Notably, early-life infection exhibits a stronger protective effect, and passive transfer of Tregs is sufficient to transfer protective immunity to uninfected mice (76). This highlights the long-term effects of post-infection immune regulation. Although early-life *H. pylori* infection confers protective effects against asthma, it is also a risk factor for gastric cancer and other diseases, warranting a risk-benefit assessment in clinical applications. Its virulence factor, VacA, may offer a safer alternative, but its efficacy as a standalone therapy requires further validation.

Microorganisms and their products can also regulate asthmatic immune responses through other mechanisms. Studies using the DMM model have shown that a transient reduction in gut microbial diversity during the neonatal period causes mice to exhibit more severe AHR and lung inflammation when exposed to HDM allergens, with this effect exhibiting persistence. The underlying mechanism is associated with an increased proportion of Th17 cells among pulmonary CD4^+^ T cells, and blocking IL-17A significantly reduces AHR (49). Microbial metabolites such as SCFA promote FoxP3 expression and Treg stability by inhibiting HDACs. In animal models of asthma, oral administration of SCFA or fiber-rich diets significantly reduces asthma symptoms and allergic inflammation (139). The prophylactic effect of whole-cell lysates of *H. pylori* (administered orally or via intraperitoneal injection) against asthma is dependent on both heat-sensitive components in the bacteria and endogenous IL-10 production by DCs (84). Additionally, various probiotics, such as *L. rhamnosus* GG, exert systemic immunomodulatory effects via the gut-lung axis, thereby improving allergic airway inflammation and asthma symptoms (140). However, clinical evidence for specific strains such as *L. rhamnosus* LGG in asthma remains limited, and their benefits in asthma prevention require further validation in large-scale studies. Furthermore, LBP, including attenuated strains of *P. aeruginosa*, engineered strains of *Mycobacterium bovis* BCG, and attenuated *Salmonella* strains, exhibit immunomodulatory potential in lung disease models through local colonization, biomolecule secretion, and immune system modulation (141). Although the potential risks of live bacteria in immunocompromised hosts, such as pathogenicity, horizontal gene transfer, and long-term perturbation of the gut or lung microbiome, have yet to be fully assessed, live attenuated strains with immunomodulatory potential remain promising as live biotherapeutic products.

### 4.2 Lung microbiome engineering and perspectives on precision medicine

Precision medicine strategies based on the lung microbiome first rely on in-depth analysis of lung microbiome heterogeneity in asthmatic patients. Studies using metagenomic sequencing have revealed that the lung microbiome of patients with severe asthma exhibits significant subtype-specific characteristics: for instance, the non-smoking severe asthma group (SAn) is enriched with *H. influenzae* and *M. catarrhalis*, while the smoking/ex-smoking severe asthma group (SAs/ex) is enriched with *H. influenzae* and *T. whipplei*. Furthermore, these microbiome characteristics exhibit a certain degree of temporal stability, providing a basis for precision stratification and targeted intervention (35). Similarly, the bronchial microbiota of patients with mild allergic asthma also exhibit distinct characteristics: for example, genera such as *Haemophilus* and *Neisseria* are enriched, while sequences of *Lactobacillales* are decreased (142). This suggests that microbiome biomarkers for different asthma phenotypes may guide the development of personalized treatment regimens.

Microbiota remodeling is one of the core strategies for regulating the lung microbiome. Targeted elimination of specific pathogens may serve as a key approach; for instance, long-term low-dose exposure to *H. influenzae* can drive the shift of the airway inflammatory phenotype toward steroid-resistant neutrophilic inflammation, accompanied by functional impairment of Tregs and impaired phagocytic capacity of pulmonary macrophages. Thus, reducing the load of such pathogens may ameliorate steroid resistance and excessive inflammation (69). However, the precise dose and duration of *H. influenzae* exposure required to drive inflammatory phenotype switching, as well as its interaction with host genetic background, remain unclear, and existing studies lack standardized models. Inhaled probiotics, as a local intervention approach, can restore airway microecological balance via immunomodulation or interbacterial competition. Meanwhile, phage therapy, due to its precise elimination capacity for specific bacterial strains, avoids the disruption of commensal microbiota by broad-spectrum antibiotics while reducing pathogen load, thereby demonstrating promising clinical translation potential (143). It should also be recognized that the airway retention, clearance, potential release of bacterial endotoxins, and off-target effects on non-target microbiota of phages require systematic investigation. Furthermore, the remote regulation of gut microbiota cannot be overlooked. *A. muciniphila* exhibits a significant reduction in abundance in patients with severe asthma, and oral supplementation of this bacterium can alleviate AHR and inflammation in mouse models, suggesting that the gut-lung axis serves as a key target for cross-organ microbiome intervention (115). The mechanisms of the gut–lung axis through which oral administration of bacteria such as *A. muciniphila* alleviates asthma symptoms warrant further investigation.

Microbial metabolite intervention represents another potential precision therapeutic approach. The bronchial microbiota of patients with mild allergic asthma exhibits enhanced metabolic functions for amino acids and SCFAs (e.g., butyrate, propionate). Meanwhile, metabolites such as SCFAs can influence airway inflammatory status by regulating immune cell function (56). Meanwhile, metabolites, vesicles, and microbial components released by gut commensal bacteria can continuously shape the immune system, providing metabolite-based intervention strategies for the prevention and treatment of respiratory diseases (144, 145). However, lung microbiome metabolic findings in asthma require validation by targeted or untargeted metabolomics. Despite the efficacy of SCFA or high-fiber diets in animal models, no randomized controlled trials of oral or inhaled SCFA have been conducted in asthmatic patients. Moreover, the complexity and interindividual variability of gut and lung microbial metabolites, influenced by diet, genetics, and medication, preclude a unified metabolite-efficacy relationship, highlighting the need for further research.

Biomarker-guided prediction of treatment response is key to achieving precision medicine. In anti-IL-13/IL-4 monoclonal antibody (mAb) therapy, patients with high Th2 inflammatory features (e.g., blood eosinophil count ≥300 cells/μL, fractional exhaled nitric oxide [FENO] ≥ 50 ppb) exhibit a more significant reduction in exacerbations. This suggests that combining microbiome characteristics with host immune biomarkers (e.g., inflammatory phenotypes, molecular phenotypes) can further optimize treatment selection (146). For instance, reduced airway microbial diversity is associated with neutrophilic asthma, while the abundance of *M. catarrhalis* is positively correlated with sputum neutrophil levels (35, 36). These microbial biomarkers may be combined with traditional biomarkers to improve the accuracy of predicting treatment response. However, current evidence linking microbial biomarkers to treatment response is largely derived from cross-sectional or retrospective analyses, lacking prospective clinical validation of their independent or combined predictive value. Moreover, most studies are limited to specific asthma phenotypes (e.g., eosinophilic or neutrophilic), with insufficient investigation into mixed granulocytic, paucigranulocytic, or post-biologic dynamic changes in the microbiome, highlighting the need for further evidence.

Future research directions should focus on multidimensional integration and technological innovation. Multi-omics analysis (integrating host, microbial, and clinical data) facilitates more refined patient stratification, drug screening, and identification of novel therapeutic targets, while defining the criteria for a “healthy” lung microbiome is a prerequisite for evaluating intervention efficacy. Early life (including the prenatal period) is regarded as a critical window for microbial interventions to prevent allergic diseases (147), suggesting that early microbiome modulation may serve as a key strategy for the primary prevention of asthma. Additionally, advances in precision microbiome engineering technologies, such as gene editing and engineered phage vectors, will promote the achievement of higher-precision microbiota regulation, providing more powerful tools for the personalized treatment of asthma.

## 5 Research methods and technical challenges

### 5.1 Technical approaches and analytical methods in lung microbiome research

Advances in second- and third-generation high-throughput sequencing have enabled detection of complex microbial communities, including unculturable microorganisms, in lung microbiome studies of asthma. Bacterial profiling commonly uses 16S rRNA hypervariable region sequencing; for example, high-throughput 16S sequencing revealed decreased airway abundance of Firmicutes and *Lactobacillus* in neonates with severe bronchopulmonary dysplasia (BPD) (148). Fungal analysis employs 18S rRNA (suitable for phylogenetics) or internal transcribed spacer (ITS) sequencing (better for genus-level differentiation) (37). Whole-genome sequencing (WGS) enables higher-resolution species identification; for instance, WGS of induced sputum from asthmatic patients using Illumina HiSeq 2500 (100 bp paired-end) and MetaPhlAn2 achieved species-level lung microbiome identification (43, 149). RNA-Seq of lung tissue identified differentially expressed genes following OM-85 treatment, and weighted gene co-expression network analysis (bWGCNA) was integrated to explore gene modules associated with asthma phenotypes (72).

Immunophenotypic and functional analyses provide direct evidence of microbe-immune crosstalk. Flow cytometry enables phenotyping of lung and draining lymph node immune cells, e.g., distinguishing dendritic cell subsets by CD11b, CD103, and CD207/langerin, and analyzing Treg phenotypes (75). And can be combined with cytokine (IL-7, IL-8) detection in BALF and blood. Immunohistochemistry visualizes lung pathology; for instance, OM-85 inhibited OVA-induced goblet cell metaplasia, and inhaled Lactobacillus biotherapeutics improved lung structure and reduced neutrophil infiltration (72, 109).Cell function assays validate microbial effects: bone marrow-derived dendritic cells (BMDCs) pretreated with OM-85 induced FOXP3^+^ Tregs upon OVA stimulation, and *Lactobacillus* components (peptidoglycan, S-layer proteins) and metabolites (L(+)-lactic acid) dose-dependently inhibited MMP-9 expression (137). *In vivo* functional evaluation often combines invasive pulmonary function measurements (e.g., AHR, lung tissue mechanics), BALF cytology, cytokine assays, and trans-epithelial electrical resistance (TEER) to assess airway epithelial barrier function. For example, evaluating the protective effect of OM-85 against Alternaria-induced barrier damage (72).

Technical challenges persist in lung microbiome research. Low-biomass samples (e.g., BALF) are prone to contamination from reagents and handling, requiring neutral models to identify oral contaminants. Differences in sampling methods (bronchial brushing, lavage fluid, lung tissue) and insufficient sequencing depth may bias community composition assessments, and the choice of 16S rRNA hypervariable regions (e.g., V1–V3 or V3–V5) can also affect diversity analyses (150, 151). All these factors need to be taken into account in experimental design.

### 5.2 Limitations of existing studies and future directions for improvement

Although the gut–lung axis has been established as a critical pathway in asthma pathogenesis, the specific associations of certain bacterial species, particularly pulmonary bacteria such as *S. pneumoniae* and *H. influenzae*, with distinct asthma subtypes have not been systematically synthesized. Moreover, heterogeneity in microbiome sampling methods, lack of standardized analytical pipelines, and insufficient validation of causality remain underappreciated.

Existing studies face multiple challenges in exploring the lung microbiome–asthma association. First, airway sampling remains difficult due to local niche differences within the upper respiratory tract (e.g., nasopharynx, even within the nasal cavity), highlighting the need for standardized sampling methods and high-resolution profiling of the upper respiratory microbiota (152–154). In contrast, due to the low bacterial density and difficulty in accessing the lower respiratory tract, relevant studies are particularly scarce (155). Moreover, invasive sampling techniques such as bronchoalveolar lavage are difficult to apply in healthy populations; consequently, most lung microbiome data are derived from cross-sectional studies of diseased individuals, limiting insights into microbial dynamics in health (156). Standardization of data analysis remains a major challenge. High inter-individual variability and inconsistent sampling methods limit cross-study comparability, underscoring the need for unified sampling standards and data integration strategies to improve reliability.

Establishing causality remains a major bottleneck; most studies only reveal correlations between microbiota composition and asthma, lacking definitive causal evidence. Model systems (e.g., animal models, airway organoids) can simulate certain physiological processes but fail to fully recapitulate the complex human ecosystem or the impact of environmental factors (157). Although stable human airway submucosal gland organoid models have been established to characterize cellular heterogeneity, inflammation, and viral tropism relative to surface airway epithelial organoids, they lack immune cells and thus cannot recapitulate native epithelial interactions with macrophages, DCs, Tregs, ILC2s, or MCs. Consequently, *in vitro* inflammatory readouts reflect only epithelial-intrinsic responses, not the full immune ecosystem. Moreover, the absence of microbiota precludes faithful modeling of the human infection milieu, and current limitations in microbial functional annotation hinder mechanistic dissection of host–microbe interactions (158, 159).

To address these challenges, future research should: First, implement longitudinal sampling to reveal host–microbe dynamics and clarify causality (160). Second, standardize sampling and data analysis to ensure cross-study comparability (161). Third, integrate multi-omics (host, microbial, clinical) and include non-bacterial communities (viruses, phages, fungi) for ecosystem-level insights (162, 163). Fourth, develop advanced models (e.g., airway chips, gene-edited organoids) combined with human challenge studies to assess single variables (e.g., co-colonizing microbes/viruses) under controlled conditions (159, 164).

## 6 Conclusion

The lung microbiome plays a pivotal role in asthma pathogenesis, progression, and inflammatory phenotype differentiation. Patients with distinct phenotypes (e.g., neutrophilic vs. eosinophilic asthma) exhibit significant differences in microbial composition, diversity, dominant taxa, and community functions. Specific pathogenic bacteria (e.g., *H. influenzae*, *M. catarrhalis*, *T. whipplei*) are closely associated with airway inflammation subtypes, disease severity, and treatment response. Moreover, early-life lung microbial colonization influences asthma risk by shaping the airway immune microenvironment via CD4^+^ T cell subsets and Tregs.

Complex cellular and molecular interactions between pulmonary immune cells and microorganisms regulate Th1/Th2/Th17/Treg differentiation and function via microbial metabolites, exosomes, and surface components. Accordingly, microbial lysates, probiotics, and their derivatives offer novel asthma therapies targeting inflammatory phenotypes and overcoming resistance. Lung microbiome-based precision medicine enables asthma phenotyping and treatment response prediction, emphasizing microbiota remodeling, metabolite intervention, and microbiome engineering in personalized therapy. Early-life microbiome modulation represents a critical prevention window, and multi-omics integration is essential to dissect microbiome–host immune crosstalk. However, challenges remain, including sampling limitations, low-biomass contamination, insufficient data standardization, and difficulty establishing causality. Future studies should incorporate longitudinal monitoring, improved sampling and analytical standards, multi-omics (metagenomics, transcriptomics, immunomics), and advanced *in vitro*/*in vivo* models to clarify lung microbiome functions and mechanisms in asthma immunopathology.

In summary, as a key participant in asthma immune regulation, the lung microbiome influences the pathological process and clinical manifestations by mediating diverse cellular and molecular pathways. In-depth understanding of its mechanisms provides a theoretical basis and technical support for asthma diagnostic phenotyping, therapeutic targeting, and disease prevention, promoting the transformation and development of pulmonary microecology towards precision medicine.

## Glossary

Th2: T helper 2
Th17: T helper 17
Tregs: Regulatory T cells
LPS: Lipopolysaccharide
MAMPs: Microbial-associated molecular patterns
PRRs: Pattern recognition receptors
IL-8: Interleukin-8
COPD: Chronic obstructive pulmonary disease
NLRP3: NLR Family Pyrin Domain Containing 3
IL-4: Interleukin-4
IL-5: Interleukin-5
IL-13: Interleukin-13
ACT: Asthma control test
HDM: House dust mite
AHR: Airway hyperresponsiveness
OVA: Ovalbumin
DMM: Delayed early microbial maturation
Th1: T helper type 1
IL-17: Interleukin-17
IL-17A: Interleukin-17A
IL-17F: Interleukin-17F
BALF: Bronchoalveolar lavage fluid
RSV: Respiratory syncytial virus
RV-A: Rhino virus A
OMVs: Outer membrane vesicles
CRP: C-reactive protein
Spls: Serine protease-like proteins
PAR-2: Protease-activated receptor-2
NF-κB: nuclear factor kappa-B
STAT3: Signal Transducer and Activator of Transcription3
STAT6: Signal Transducer and Activator of Transcription6
JAK-STAT: Janus kinase-signal transducer and activator of transcription
MAPK: Mitogen-Activated Protein Kinase
PI3K: Phosphatidylinositol 3-kinase
Nrf2: Nuclear factor erythroid 2-related factor 2
ILC2s: Group 2 innate lymphoid cells
MCs: mast cells
TSLP: Thymic Stromal Lymphopoietin
IL-25: Interleukin-25
IL-33: Interleukin-33
IL-1β: Interleukin-1β
TNF-α: Tumor Necrosis Factor-α
IFN-γ: Interferon gamma
IL-6: Interleukin-6
IL-18: Interleukin-18
SEs: *Staphylococcus aureus* enterotoxins
ECP: Eosinophil cationic protein
NETs: Neutrophil extracellular traps
AECs: Airway epithelial cells
ECs: Epithelial cells
Th9: T helper type 9 cell
Tfh: Follicular helper T cell
DCs: Dendritic cells
T_N_: naive T cells
IL-9: Interleukin-9
IL-22: Interleukin-22
IL-23: Interleukin-23
TGF-β: Transforming growth factor-β
CXCL1: chemokine C-X-C motif ligand 1
SCFAs: Short-chain fatty acids
HDACs: Histone deacetylases
MDSCs: Myeloid-derived suppressor cells
IL-10: Interleukin-10
Arg1: Arginase 1 iNOS, Inducible nitric oxide synthase
IDO: Indoleamine 2,3-dioxygenase
ILC3s: Group 3 innate lymphoid cells
TLR4: Toll-like receptor 4
TLR5: Toll-like receptor 5
TLR13: Toll-like receptor 13
TLR8: Toll-like receptor 8
TLR2: Toll-like receptor 2
MD2: Myeloid Differentiation Protein-2
BEVs: Bacterial exosomes
EVs: Extracellular vesicles
LTA: Lipoteichoic acid
CPS: Cell wall polysaccharides
NOD: Nucleotide-binding oligomerization domain
NLRs: Nod-like receptors
NTHi: Non-typable *Haemophilus influenza*
PBL: Polyvalent bacterial lysates
PABL: polyvalent alkaline bacterial lysate
PMBL: Polyvalent mechanical bacterial lysates
GGT: γ-glutamyl transferase
LGG: *L. Rhamnosus* gg
BPD: Bronchopulmonary dysplasia
MMP-9: Matrix metalloproteinase-9
NOD2: Nucleotide-binding oligomerization domain 2
moDCs: Monocyte-derived dendritic cells
NODs: Nucleotide-binding oligomerization domain receptors
FlaB: Flagellin b
rDCs: Regulatory dendritic cells
LBP: Live biotherapeutic product
SAn: Non-smoking severe asthma group
SAs/ex: Moking/ex-smoking severe asthma group
mAb: Monoclonal antibody
FENO: Fractional exhaled nitric oxide
ITS: Internal transcribed spacer
WGS: Whole-genome sequencing
RNA-Seq: Rna sequencing
bWGCNA: Weighted gene co-expression network analysis
TAC: Transcriptome-associated clusters
IHC: Immunohistochemical
BMDCs: Bone marrow-derived dendritic cells
TEER: Trans-epithelial electrical resistance.
